# A/C magnetic hyperthermia of melanoma mediated by iron(0)/iron oxide core/shell magnetic nanoparticles: a mouse study

**DOI:** 10.1186/1471-2407-10-119

**Published:** 2010-03-30

**Authors:** Sivasai Balivada, Raja Shekar Rachakatla, Hongwang Wang, Thilani N Samarakoon, Raj Kumar Dani, Marla Pyle, Franklin O Kroh, Brandon Walker, Xiaoxuan Leaym, Olga B Koper, Masaaki Tamura, Viktor Chikan, Stefan H Bossmann, Deryl L Troyer

**Affiliations:** 1 Department of Anatomy and Physiology, 228 Coles Hall, Kansas State University, Manhattan, KS 66506, USA; 2 NanoScale Corporation, 1310 Research Park Drive, Manhattan, KS 66502, USA; 3 Department of Chemistry, 213 CBC Building, Kansas State University Manhattan, KS 66506, USA

## Abstract

**Background:**

There is renewed interest in magnetic hyperthermia as a treatment modality for cancer, especially when it is combined with other more traditional therapeutic approaches, such as the co-delivery of anticancer drugs or photodynamic therapy.

**Methods:**

The influence of bimagnetic nanoparticles (MNPs) combined with short external alternating magnetic field (AMF) exposure on the growth of subcutaneous mouse melanomas (B16-F10) was evaluated. Bimagnetic Fe/Fe_3_O_4 _core/shell nanoparticles were designed for cancer targeting after intratumoral or intravenous administration. Their inorganic center was protected against rapid biocorrosion by organic dopamine-oligoethylene glycol ligands. TCPP (4-tetracarboxyphenyl porphyrin) units were attached to the dopamine-oligoethylene glycol ligands.

**Results:**

The magnetic hyperthermia results obtained after intratumoral injection indicated that micromolar concentrations of iron given within the modified core-shell Fe/Fe_3_O_4 _nanoparticles caused a significant anti-tumor effect on murine B16-F10 melanoma with three short 10-minute AMF exposures. We also observed a decrease in tumor size after intravenous administration of the MNPs followed by three consecutive days of AMF exposure 24 hrs after the MNPs injection.

**Conclusions:**

These results indicate that intratumoral administration of surface modified MNPs can attenuate mouse melanoma after AMF exposure. Moreover, we have found that after intravenous administration of micromolar concentrations, these MNPs are capable of causing an anti-tumor effect in a mouse melanoma model after only a short AMF exposure time. This is a clear improvement to state of the art.

## Background

Recently, questions have emerged regarding whether anticancer drug development is headed in the right direction and whether opportunities that are off the accepted path are being overlooked [1]. Largely due to the increasing insight into the series of mutations associated with the development of cancer, drug development has moved into the "molecular target" area. There have been initial successes (e.g. imatinib mesylate for the treatment of chronic myelogenous leukemia and gastrointestinal stromal tumors); however, the genetic complexity and diversity of tumor cells, including the occurrence of cancer stem cells, have prevented molecular targeting from becoming universally successful. Because the progression of a normal cell to a cancer cell involves numerous genetic mutations, targeting one or even several gene products may be ineffective. Furthermore, many biological processes feature alternate pathways which can be upregulated, if needed, thus thwarting molecularly targeted therapies [1]. To overcome these obstacles, a successful cancer therapy has to combine several approaches. Molecular targeting can be a viable component of this methodology. However, other approaches, such as stem cell delivery, hyperthermia, photodynamic therapy, and the design of multifunctional platforms that combine cancer diagnostics and treatment (theranostics) have not received full attention during the last decade.

We and others are working toward cost-effective treatment methods based on non-conventional combinations of known and proven techniques. We asked whether it is possible to achieve cancer localization by using porphyrin labels for the delivery of iron-containing superparamagnetic nanoparticles to tumor tissue. Tumor cells selectively uptake porphyrins, which they need as prosthetic groups in their elevated sugar metabolism, via over-expression of porphyrin receptors in their cell membranes [2]. There is a strong positive correlation between the cell uptake of a variety of chemically defined, synthetic and natural porphyrins and their octanol/water distribution coefficients [3,4]. These findings support the paradigm that there indeed exists a porphyrin uptake mechanism other than endocytosis in cancer cells. The LDL (lowdensity lipoprotein) receptor, which is over-expressed in cancer cells, has the ability to take up porphyrins as well, either alone or simultaneously with other porphyrin receptors. Localized hyperthermia is a powerful therapeutic modality. When administered selectively, hyperthermia treatment can be very potent against many types of cancer because it is not based on the intake of drugs by cancer cells, but on the application of heat. When heated to 45°C, vital proteins of the cancer cell become damaged (*e.g. *misfolded) and/or the cell membrane partially dissolves in the surrounding aqueous medium [5]. A multitude of heat-induced deviations from the "normal" metabolism of a cancer cell can eventually lead to apoptosis (programmed cell death). Although many cancer types are slightly more susceptible to hyperthermia than healthy cells, the latter essentially share the same fate when heated [6]. Therefore, the development of methods to localize hyperthermia to cancer cells remains one of the challenges in this field. This is important when attempting to treat solid tumors within the human body as well as for treatment of metastasizing cancers. The use of tethered porphyrins as "bait" may provide an effective (and low cost) alternative to using antibodies for getting magnetic nanoparticles effectively into tumors, which is currently a common tumor targeting method.

Magnetic nanoparticles dump thermal energy into the system, therefore providing heating (magnetic hyperthermia) [7]. Heating takes place by power absorption of magnetic particles due to an A/C magnetic field [8]. The important factor for magnetic heating experiments is the specific absorption rate (SAR), which is determined by SAR = C × ΔT/Δt, where C is the specific heat capacity of the sample and T and t are the temperature and time, respectively. SAR is very sensitive to the material properties. While in multi-domain particles the dominant heating is hysteresis loss due to the movement of domain walls, this is not the case with small, single domain particles. The two main contributing mechanisms of SAR in single domain magnetic NPs are the Brownian (rotation of the entire nanoparticle) and Néel (random flipping of the spin without rotation of the particle) relaxations [9,10]. The transition between the two mechanisms occurs between 5-12 nm for various materials, but it also varies with frequency [11]

Melanoma incidence has reached almost epidemic proportions worldwide. When it is present as disseminated metastatic disease it is not curable using current clinical tools; traditional chemotherapy is often ineffective due to inherent drug resistance [12]. We have selected the B16-F10 melanoma model in syngeneic C57BL/6 female mice because this type of melanoma has proven to be quite resistant to treatment. In order to evaluate the efficacy of hyperthermia treatment against cancer, we did not want to select a less resistant cancer cell line/host system. In addition, since hyperthermia often enhances the immune response, an immunocompetent host was desirable.

In the work reported here, we have used (bi)magnetic iron/iron oxide core/shell nanoparticles, synthesized by NanoScale Corporation (Manhattan, KS), for A/C (alternating current)-magnetic cancer therapy, because they exhibit superior properties in several areas: The nanoscopic size (d<15 nm) of the stealth-protected Fe/Fe_3_O_4 _core/shell nanoparticles will permit passive tumor targeting from the bloodstream by using the EPR (enhanced permeation and retention) effect [13,14]. In addition, we surmise that our porphyrin coating may enhance MNP uptake in tumors, because cancer cells are in constant need of porphyrins as prosthetic groups in their elevated sugar metabolism [2] and overexpress the LDL(low-density lipoprotein) receptor. This remains an area for future clarification. Here, we compared the effects of intratumoral (IT) and intravenous (IV) core/shell porphyrin-tethered nanoparticle treatment followed by A/C exposure on B16-F10 melanoma growth in a mouse model.

## Methods

### Cell lines and animals

B16-F10 melanoma cells were purchased from ATCC (Manassas, VA) and maintained in DMEM (Dulbecco's Modified Eagle Medium) supplemented with 10% fetal bovine serum (FBS) and 1% penicillin-streptomycin in a humidified 37°C incubator at 5% CO_2_.

6-8 week old female C57/BL6 mice were purchased from Charles River Laboratories (Wilmington, MA). Mice were maintained according to approved institutional IACUC guidelines in the Comparative Medicine Group Facility of Kansas State University. All animal experiments were conducted according to these IACUC guidelines.

### Transmission Electron Microscopy

The sizes of the different nanoparticles were determined by using TEM. This was achieved employing a Philips CM-200 TEM instrument operating at 100 kV. 1-2 micrograms of the MNPs were dissolved in anhydrous tetrahydrofuran THF (5 mL) and one drop of the resulting nanoparticle solution was spread over a copper grid (300 mesh size) supporting a thin film of amorphous carbon. To reduce the damage from the electron beam, the sample was cooled to liquid nitrogen temperature during data collection.

### Porphyrin-tethered Stealth-Coated (Bi) Magnetic Fe/Fe_3_O_4 _Nanoparticles

**Figure 1:**
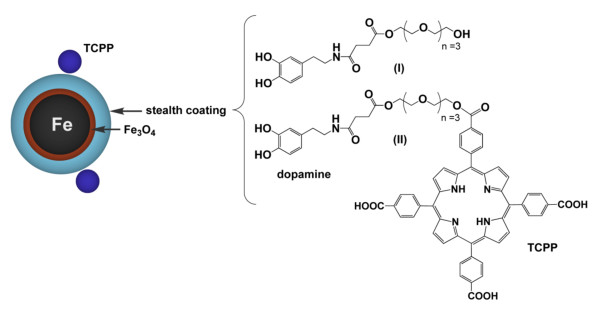
**Composition of the 4-tetracarboxyphenyl porphyrin (TCPP)-labeled, dopamine-anchored tetraethylene glycol ligands**. Nanoparticles of 7.2 nm (outer diameter) require approximately 120 dopamine-anchored ligands (assuming a monolayer).

**Figure 2:**
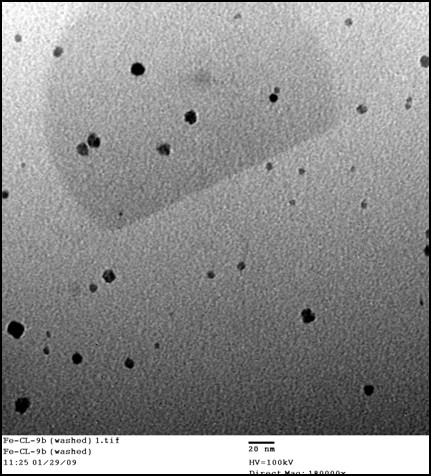
**TEM image of Fe/Fe_3_O_4 _core/shell nanoparticles featuring an organic protective dopamine-anchored stealth layer**.

The synthesis of the stealth-coated dopamine-labeled Fe/Fe_3_O_4 _nanoparticles featuring tethered 4-tetracarboxyphenyl porphyrins (TCPP) is reported in a separate publication (Wang *et al.*, ACS Nano, 2010, in preparation). Briefly, Fe/Fe_3_O_4_-core/shell nanoparticles were synthesized by NanoScale Corporation and then coated with dopamine-anchored ligands. The structure of the nanoparticles is shown in Figure 1, and results from electron microscopy are shown in Figure 2. The diameter of the Fe(0)- core was 5.4 ± 1.1 nm; the diameter of the inorganic Fe/Fe_3_O_4_-nanoparticles was determined to be 7.2 ± 2.8 nm. Using the program IMAGE (NIH), we have determined the polydispersity index of the Fe/Fe_3_O_4_-nanoparticles to be 1.31. Note that the stealth ligand has a length of 2.5 nm (AM1-Chemdraw Ultra 3D package, Cambridge Soft Corporation, Cambridge, MA 02140), so that the resulting bimagnetic nanoparticles are 12 ± 3 nm in size. The porphyrin-labels have a diameter of 1.95 nm (AM1). Note that the dopamine-anchored tetraethylene glycol ligand (I) and the TCPP-linked dopamineanchored tetraethylene glycol ligand (II) have been synthesized separately. The binding of the ligands to the Fe_3_O_4 _layer was achieved in anhydrous THF under argon; the molar ratio of ligands I/II was 100/4. The reaction procedure is described in detail in a separate paper (Wang *et al.*, ACS Nano, 2010, in preparation). We assume a statistical distribution of the ligands at the Fe_3_O_4 _surface. Assuming a Poisson distribution, 96.4 percent of the Fe/Fe_3_O_4 _NPs at the chosen ratio feature at least one chemically linked TCPP unit, which will act as "bait" for the B16-F10 cancer cells. The solubility of the organically coated Fe/Fe_3_O_4 _NPs was determined to be 0.35 mg ml^-1 ^and the Specific Adsorption Rate (SAR) at the field conditions described here was 64 ± 2 Wg^-1 ^(Fe).

### Determination of iron concentration in MNPs

Iron concentration in MNPs was measured using the Ferrozine-based spectrophotometric iron estimation method [15]. For this method, 50 μl of MNPs were diluted to 1 ml with distilled water. MNPs were then lysed by incubating for 2 hours at 65-70°C after the addition of 0.5 ml of 1.2 M HCl and 0.2 ml of 2 M ascorbic acid. After incubation, 0.2 ml of reagent containing 6.5 mM Ferrozine, 13.1 mM neocuproine, 2 M ascorbic acid, and 5 M ammonium acetate was added and incubated for 30 minutes at room temperature. After 30 minutes, the optical density of the samples was measured using a UV-VIS spectrophotometer at 562 nm. A standard curve was prepared using 0, 0.1, 0.2, 0.5, 1, 2, 5 μg/ml ferrous ammonium sulfate samples. Water with all other reagents is used as blank.

### Magnetic Heating

The nanoparticles used in these experiments are dominated by Néel relaxation due to the superparamagnetic nature of the iron(0) cores. The hyperthermia apparatus (Superior Induction Company, Pasadena, CA) used here has a "heavy duty" induction heater converted to allow measurement of the temperature change of a sample. In the setup, a remote fiber optic probe (Neoptix, Quebec, Canada) is used to monitor the temperature change. The frequency is fixed (366 kHz, sine wave pattern); field amplitude is 5 kA/m. The coil diameter is 1 inch, 4 turns continuously water cooled. For all *in vivo *experiments, the mice were placed into the induction coil using a specially designed Teflon supporter so that tumors were located exactly in the region of the AMF possessing the highest field density.

### Cytotoxicity of Magnetic Nanoparticles on B16-F10 cells

Potential cytotoxic effects of MNPs were studied by incubating cells in differing concentrations of MNPs. B16-F10 cells were incubated overnight with MNPs amounts corresponding to 5, 10, 15, 20, and 25 μg/mL iron. After incubation, the medium was removed and the cells were washed twice with DMEM and cells were counted via hemocytometer with Trypan blue staining. This method also allows counting non-viable cells since only they allow the blue stain into the cell. All experiments were run in triplicate and repeated at least twice.

### Temperature measurements on mice

MNPs containing 100 μg of iron in 100 μl of distilled water were injected into the rear limb muscle of one mouse and the leg was then exposed to AMF for 10 min. An optical temperature probe was inserted intramuscularly at the injection site and the temperature increase was measured during AMF exposure. At the same time, the body temperature was monitored with a separate temperature probe.

### Intratumoral Hyperthermia

Ten mice were transplanted subcutaneously into each rear limb above the stifle with 1 × 10^6 ^B16-F10 melanoma cells suspended in PBS. 120 μL of saline were injected into melanomas on the left leg of all mice and 120 μl MNPs containing 1 mg Fe/mL were injected into right leg tumors of all mice in three injections on day 4, 5, 6 (total of 360 μg iron). Because the MNPs were dilute and tumor volumes were quite small, 3 injections instead of 1 were needed in order to reach this level of MNPs. Both left (saline) and right (MNPs) leg tumors of five of the mice were exposed to AMF for 10 minutes soon after injections. Tumors on the remaining five mice were not exposed to AMF. Based on this, there were 4 groups which tested the effects of MNPs with and without AMF and of AMF alone: Group 1: Intratumoral saline injection, not exposed to AMF (left legs of first five mice); Group 2: Intratumoral injection of saline, exposed to AMF (left legs of remaining five mice); Group 3: Intratumoral injection of MNPs, not exposed to AMF (right legs of first five mice); Group 4: Intratumoral injection of MNP, exposed to AMF (right legs of remaining five mice). After three AMF exposures, tumor sizes were measured with a caliper on days 8 to 14, and tumor volume was calculated using the formula 0.5aXb^2 ^(a = longest diameter; b = smaller diameter). After 14 days mice were euthanized, tumors were excised, and tumor weights were measured.

### Intravenous administration of MNPs with AMF exposure

On day 0, 0.35 × 10^6 ^B16-F10 melanoma cells were injected subcutaneously into the right legs of 27 mice. Mice were randomly divided into three groups: Group 1, IV MNPs, no AMF; Group II, IV MNPs, AMF; Group III, DMEM control, no AMF. On days 6, 9, and 11 after tumor cell transplant, MNPs corresponding to 226 mcg of iron were injected intravenously into each mouse in groups I and II. On the same day, DMEM was injected intravenously into group III. For group II, tumors were exposed to AMF for 10 minutes one day after each I.V. MNPs injection (total of three AMF treatments). MNPs not coated with porphyrins were not sufficiently soluble in water, so we were not able to use them as control. Tumor sizes were measured using a caliper on days 14 and 18, and tumor volume was calculated as described above. On day 18 all mice were euthanized, tumors were excised, and tumor weights were measured.

### Histological Analysis

After euthanizing mice, lung, liver, and tumors were collected and snap frozen. 8-10 μm sections were made in a cryostat (Leitz Kryostat 1720). Staining for iron content on these sections was carried out by using Perl's Prussian blue staining kit (Polysciences, Inc., Warrington, PA). Apoptosis was evaluated using a DeadEnd Fluorometric TUNEL kit. (Promega Corp., Madison, WI) following the manufacturer's instructions.

### Statistical analysis

Statistical analyses were performed by Macanova 4.12 (School of Statistics, University of Minnesota, Minneapolis, MN). The means of the experimental groups were evaluated to confirm that they met the normality assumption. To evaluate the significance of overall differences in tumor volumes and tumor weights between all *in vivo *groups, statistical analysis was performed by analysis of variance (ANOVA). A p-value less than 0.1 was considered as significant. Following significant ANOVA, post hoc analysis using least significance difference (LSD) was used for multiple comparisons. Significance for post hoc testing was set at p < 0.1. All the tumor volumes and weight data were represented as mean +/- standard error (SE) on graphs.

## Results

### Cytotoxicity of Magnetic Nanoparticles on B16-F10 cells

**Figure 3:**
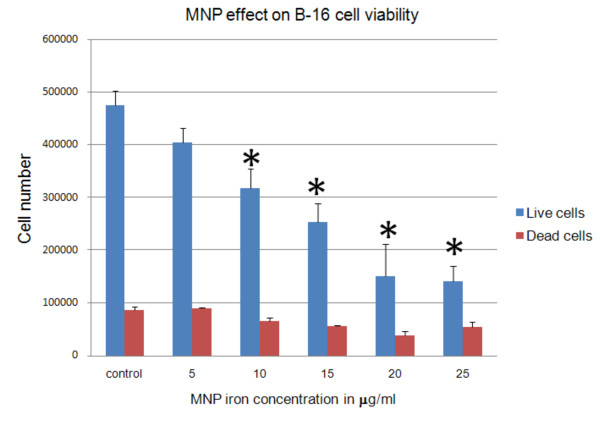
***In vitro *cell viability of B16-F10's cultured in medium containing increasing concentrations of MNPs, as measured by iron concentration**. *Statistically significant (pvalue less than 0.05).

MNP toxicity was tested after overnight incubation of B16-F10 cell cultures in 24-well plates with various MNPs concentrations, as measured by iron concentration. There was a dose-dependent cytotoxicity of the MNPs. B16-F10 cancer cell viability assessment in the presence of varying concentrations of MNPs is shown in Figure 3. The MNPs showed a pronounced cytotoxic effect on B16-F10 cells at >10 μg iron levels. (p-value < 0.05)

### Temperature measurements on mice after intramuscular MNPs injection

**Figure 4:**
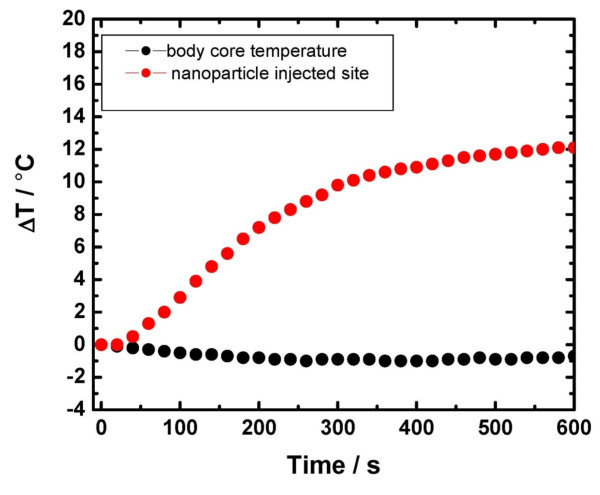
**Graph depicting temperature change at MNP injection site and in body core during AMF exposure, measured with a fiber optic temperature probe**.

We observed 11°C temperature increase subcutaneously at the MNPs injection site within 10 minutes of AMF exposure. There was no increase in core body temperature (Figure 4). These data demonstrate specific magnetic hyperthermia. However, we did not directly measure temperature change in tumors during experiments because the necessary skin opening for the probe caused leakage of the gelatinous melanoma tumor parenchyma, introducing increased potential variability in tumor volumes.

### Intratumoral Magnetic Hyperthermia

**Figure 5:**
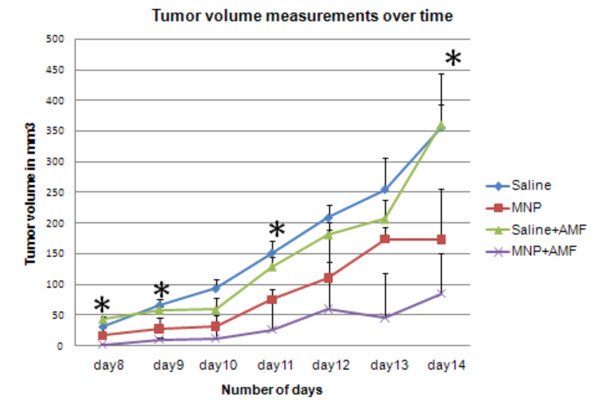
**Effect on tumor burden of intratumoral injection of MNPs followed by alternating magnetic field (AMF) treatments**. Graph depicting average tumor volumes over time of B16-F10 tumor bearing mice which were later injected with either saline or MNP intratumorally and with or without AMF treatments. *Statistically significant (pvalue less than 0.1).

After three AMF exposures, tumor sizes were measured from days 8 to 14; the comparison is shown in Figure 5. We identified decreased tumor size both in tumor-bearing mice treated with MNPs and with MNPs+AMF. The tumors with MNPs+AMF showed a significant reduction in tumor volume at 8, 9, 11, & 14 days (p < 0.1) compared to the saline treated group. A decrease in size with only MNP treatment (no AMF) relative to the saline controls was also noted; however, this decrease was not significant. Since earlier intramuscular injections and optical probe measurements revealed hyperthermia after AMF, the probable cause for the tumor attenuation shown here is local hyperthermia.

### Intravenously administered MNPs and AMF exposure

**Figure 6:**
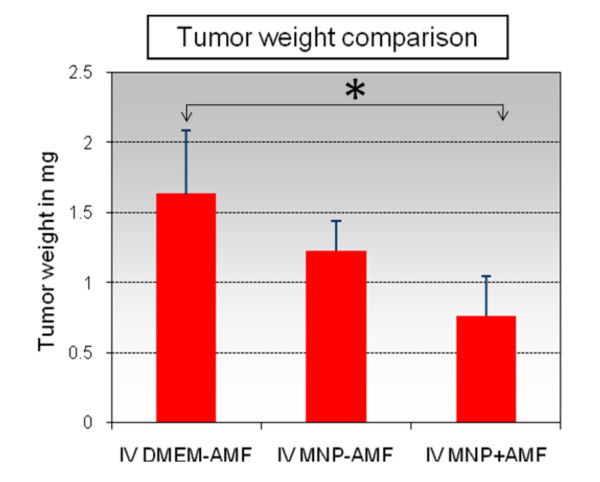
**Effect of intravenous injection of MNP and AMF on tumor weight**. *Statistically significant difference (p-value less than 0.1) between control and IV MNP+AMF groups.

**Figure 7:**
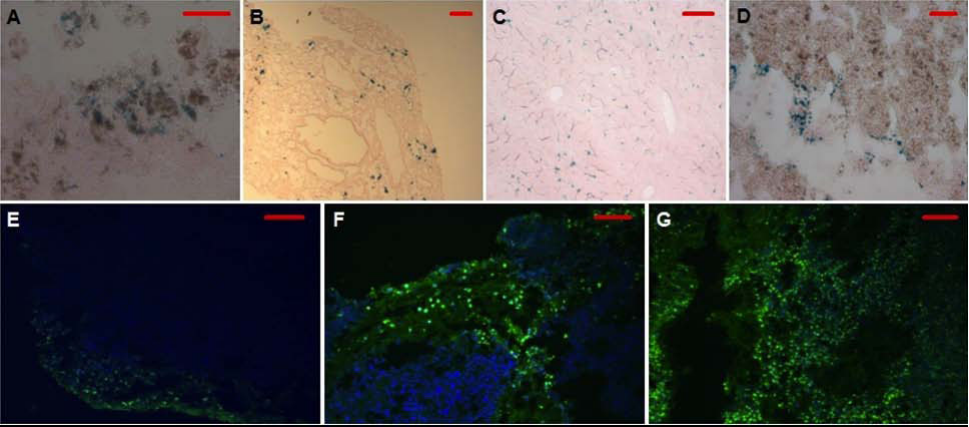
**Prussian blue staining and evaluation of apoptosis of tissues after the *in vivo *experiment**. Green fluorescence indicates apoptosis positive and blue is DAPI counterstaining (E-G). A-C: IV MNP + AMF in tumor, lung, and liver, respectively. D: Intratumoral MNP, tumor section. E-G: Apoptosis assay pictures. E: Control tumor section. F: Tumor section with intravenous MNP administration followed by AMF. G: Tumor section with intratumoral MNP administration followed by AMF. (Scale bar = 100 *μ*m) Additional file 1 Title: Tumor volume measurements on day14 and 18 of intravenously administered MNPs and AMF exposure experiment. Description: Day 14 and 18 tumor volumes of individual groups are compared. (not significant)

Tumor-bearing mice with intravenously injected MNPs were exposed to AMF treatments three times and after 18 days were euthanized. Tumor weights were obtained and compared to controls (Figure 6). A significant decrease in tumor weight (p < 0.1) was observed in the intravenous MNPs+AMF group and was most likely due to heat generated from MNPs in tumors, based on earlier optical probe experiments in anesthetized mice. Some tumor weight decrease was also observed in intravenous MNPs without AMF treatment. On days 14 and 18, tumor volumes were recorded and were markedly attenuated in the mice with MNPs with AMF; however this was not significant (Additional file 1). After tumors were harvested and sectioned, MNPs in tumor sections and other tissues were identified as Prussian blue positive material in tumor bearing mice intravenously injected with MNPs (Figure 7A-D).

### Apoptosis assay

Histological analysis after apoptosis assay with the modified TUNEL assay showed the most apoptotic positive cells in the intratumoral MNP+AMF treatments (Figure 7G), intermediate apoptosis levels in mice that received intravenous MNP+AMF (Figure 7F), and the fewest apoptotic cells in the saline+AMF group (Figure 7E).

## Discussion

The major finding of this study was that there is a significant decrease in tumor size after systemic (intravenous) administration of low (microgram iron content) amounts of the porphyrin-tethered MNPs and AMF exposure compared to tumors in animals given intravenous DMEM. We have found the MNPs in the melanomas, leading us to surmise that the porphyrins attached to them may facilitate MNP uptake. However, further studies are needed to confirm the porphyrin-facilitated MNP uptake in tumors. As already discussed, cancer cells over-express porphyrin receptors, because they require more porphyrins as prosthetic groups in their elevated sugar metabolism than normal cells [2]. Tumor localization of MNPs by urokinase plasminogen activator receptor (uPAR) is reported by Yang *et al.*[16].

Our intratumoral magnetic hyperthermia results showed that microgram amounts of iron delivered by the core-shell Fe/Fe_3_O^4 ^nanoparticles caused an antitumor effect on melanoma with short-time AMF exposures (10 min.). This is a clear improvement with respect to current protocols, which are defined by milligram amounts of MNPs and much longer exposure times, usually 30 minutes [17]. We have also observed a trend toward decreased tumor size after MNP administration without AMF exposure. This is not surprising, since our *in vitro *work indicated that the MNPs have a pronounced cytotoxic effect on B16-F10 cells. In *in vitro *studies, we used a total of 10 μg of ironcontaining MNP (in 1 ml of medium) for 500,000 cells. So theoretically, each cell is exposed to 0.00002 μg of iron. However, *in vivo*, a detectable, three-dimensional tumor contains many more cells, and the tumor interstitium offers a vastly different microenvironment than the monolayer of cells. A tumor volume of 1 cm^3 ^contains approximately 10^9 ^cells [18]. In the *in vivo *studies, we injected a total of 360 μg of ironcontaining MNPs intratumorally when mean tumor size was about 50 mm^3 ^(an estimated 50 million cells). Extrapolating our *in vitro *results, 1000 μg of iron-containing MNPs would be required for similar toxicity to 50 million cells. Therefore, we feel that the effective amount of core/shell MNPs in the tumor on a per cell basis was actually much less than in the *in vitro *studies. Minamimura *et al. *gave intratumoral iron oxide MNP injections and noted that their MNPs alone exerted a noticeable anti-melanoma effect [19]. In the study reported here, the effect of MNPs alone is most probably due to the biocorrosion of the MNPs and the subsequent release of iron(II) and iron(III) cations, which is known to cause cell damage via iron(II/III)-enhanced chemistry of reactive oxygen species (ROS) [20]. Thus, we propose a two-pronged effect: magnetic hyperthermia and the additional generation of free radicals by manifold Fenton-type reactions [20].

It must be noted that we have found significant amounts of iron in lungs and liver, as indicated by Prussian blue staining (Figure. 7B and 7C). Despite this widespread distribution of MNPs *in vivo*, we emphasize that there were no fatalities due to the blocking of arteries or exposure to AMF. This may be because the AMF was only applied to the melanoma region, preventing unwanted hyperthermia in other tissues. Our findings of minimal side effects after systemic administration of MNPs are corroborated by other work testing superparamagnetic NP for MRI capability and possible toxic effects. Wiegand *et al. *showed that 250 or 500 nm ferrofluid given intravenously to normal rabbits resulted in normal serum iron and enzymes for liver and kidney function at 1-72 hours after administration [21]. Kim *et al. *showed that silica over coated magnetic nanoparticles of 50 nm size did not cause apparent toxicity or alter the blood-brain barrier in mice after four weeks [22]. Pharmacokinetic data of the dependence of iron or iron oxide nanoparticles on their respective sizes are not yet available. Therefore, we have to rely on the available data on the distribution of stealthcoated Au-NPs (sizes: 15 nm, 50 nm and 160 nm) in rats and rabbits [23]. The larger the diameter of the Au-NPs, the higher their concentration in liver and spleen 24 h after IV-injection. The smallest Au-NPs did more evenly distribute within the mammalian bodies. Furthermore, they remained in the bloodstream distinctly longer. A longer residence time of our stealth Fe/Fe_3_O_4 _nanoparticles (diameter including the stealth ligands: 12 ± 3 nm) in the bloodstream may enhance the EPR effect (enhanced permeation and retention).

## Conclusion

The findings reported here indicate that ligand modified nanoparticles given systemically or intratumorally at low concentrations can significantly attenuate subcutaneous B16-F10 tumors in mice after repetitive short AMF exposure. Hence, it may be possible to exploit upregulated porphyrin uptake by cancer cells to facilitate getting core/shell bimagnetic nanoparticles to them, but further work is required to confirm this. After exposure to an AMF, which itself causes no harm, localized hyperthermia of cancer tissue attenuates the tumor without the undesirable side effects associated with traditional chemotherapy. This approach also circumvents failure of molecularly-targeted approaches due to redundant systems and failure of chemotherapeutic approaches due to cancer cell multidrug resistance. Furthermore, we surmise that AMF treatment is augmented by the release of iron within the tumor regions due to biocorrosion, increasing the intratumoral concentration of reactive oxygen species. Thus, localized hyperthermia after systemic administration of porphyrin labeled stealth MNPs may hold promise for future clinical therapy of melanomas. Our future goals are to further modify the MNPs to improve accumulation in tumors relative to other tissues and organs, and to test these improved MNPs in treatment of a preclinical metastatic model.

## Abbreviations

MNPs: Magnetic Nanoparticles; TCPP: 4-tetracarboxyphenyl porphyrin; AMF: Alternating magnetic field; SAR: Specific absorption rate; IT: Intratumoral; IV: Intravenous.

## Competing interests

Franklin O. Kroh, Brandon Walker, Olga B. Koper, and Xiaoxuan Leaym are full time employees at NanoScale Corp.,

## Authors' contributions

SB performed *in vitro *viability studies, *in vivo *experiments; RR designed experiments and performed *in vivo *and histology experiments; HW synthesized dopamine PEG coatings and attached porphyrins to core/shell MNPs; TS synthesized Dopamine PEG coatings and attached porphyrins to core/shell MNPs; RD performed AMF experiments; MP provided study materials for *in vitro *and *in vivo *experiments and reviewed the manuscript; FK designed core/shell MNPs; BW designed MNPs and aquired TEM; XL designed core/shell MNPs; OK designed core/shell MNPs; MT reviewed manuscript; VC supervised AMF experiments, interpreted AMF data, and reviewed the manuscript; SHB designed and supervised nanoparticle coatings synthesis, interpreted data, and reviewed the manuscript; DT designed and supervised *in vitro and in vivo *experiments, interpreted data, and reviewed the manuscript. All authors have read and approved the final manuscript.

## Pre-publication history

The pre-publication history for this paper can be accessed here:

http://www.biomedcentral.com/1471-2407/10/119/prepub

## Supplementary Material

Additional file 1 **Tumor volume measurements on day14 and 18 of intravenously administered MNPs and AMF exposure experiment**. Day 14 and 18 tumor volumes of individual groups are compared. (not significant). Click here for file
