# Supplementary figures and images for: A/C magnetic hyperthermia of melanoma mediated by iron(0)/iron oxide core/shell magnetic nanoparticles: a mouse study
Source: BMC Cancer. 2010 Mar 30;10:119. doi: 10.1186/1471-2407-10-119 (PMC2859385; doi:10.1186/1471-2407-10-119)

**Tumor volume measurements over time**

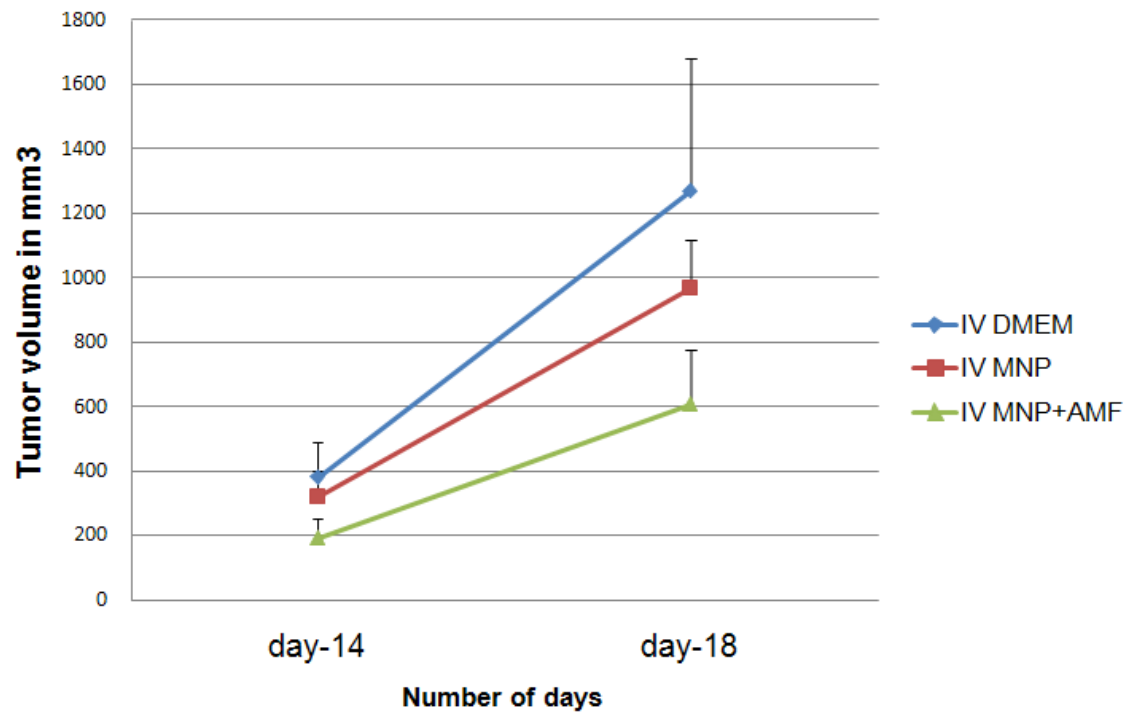

Supplement: Additional file 1 — Tumor volume measurements on day14 and 18 of intravenously administered MNPs and AMF exposure experiment. Day 14 and 18 tumor volumes of individual groups are compared. (not significant). [file 1471-2407-10-119-S1.PDF]
